# [1,4-Bis(di­phenyl­phosphan­yl)butane-κ^2^ *P*,*P*′]di­bromido­palladium(II)

**DOI:** 10.1107/S1600536814000774

**Published:** 2014-01-18

**Authors:** Kwang Ha, Yo Soon Song

**Affiliations:** a School of Applied Chemical Engineering, The Research Institute of Catalysis, Chonnam National University, Gwangju 500-757, Republic of Korea

## Abstract

In the title complex, [PdBr_2_(C_28_H_28_P_2_)], the Pd^II^ ion has a distorted *cis*-Br_2_P_2_ square-planar coordination geometry defined by two P atoms from the chelating 1,4-bis­(di­phenyl­phosphan­yl)butane ligand and two Br^−^ anions. The four phenyl rings are inclined to the least-squares plane of the PdBr_2_P_2_ unit [maximum deviation = 0.1294 (7) Å], making dihedral angles of 66.3 (2), 87.2 (2), 68.8 (2) and 86.8 (2)°. The butyl­ene chain is in a *gauche* conformation, with a C—C—C—C torsion angle of 57.0 (8)°. Inter­molecular C—H⋯Br hydrogen bonds link the complex mol­ecules into supra­molecular layers in the *ab* plane. Weak π–π inter­actions, both intra- and inter­molecular [shortest inter-centroid distance = 4.598 (5) Å], are also noted in the three-dimensional architecture.

## Related literature   

For the crystal structures of related [*M*Cl_2_(dppb)] complexes where *M* = Pd or Pt, and dppb = 1,4-bis­(di­phenyl­phosphan­yl)butane), see: Makhaev *et al.* (1996 ▶); Deacon *et al.* (2005 ▶).
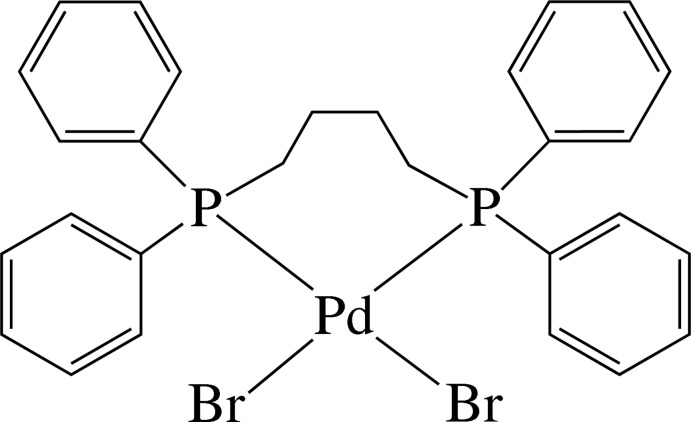


## Experimental   

### 

#### Crystal data  

[PdBr_2_(C_28_H_28_P_2_)]
*M* *_r_* = 692.66
Triclinic, 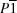

*a* = 8.7481 (5) Å
*b* = 10.7677 (6) Å
*c* = 14.4789 (8) Å
α = 87.203 (1)°
β = 79.389 (1)°
γ = 73.929 (1)°
*V* = 1288.15 (13) Å^3^
*Z* = 2
Mo *K*α radiation
μ = 3.96 mm^−1^
*T* = 200 K
0.06 × 0.04 × 0.01 mm

#### Data collection  

Bruker SMART 1000 CCD diffractometer
Absorption correction: multi-scan (*SADABS*; Bruker, 2000 ▶) *T*_min_ = 0.859, *T*_max_ = 1.000
8009 measured reflections
4961 independent reflections
3847 reflections with *I* > 2σ(*I*)
*R*_int_ = 0.024

#### Refinement  

*R*[*F* ^2^ > 2σ(*F* ^2^)] = 0.045
*wR*(*F* ^2^) = 0.120
*S* = 1.08
4961 reflections
298 parameters
H-atom parameters constrained
Δρ_max_ = 1.13 e Å^−3^
Δρ_min_ = −0.96 e Å^−3^

### 

Data collection: *SMART* (Bruker, 2000 ▶); cell refinement: *SAINT* (Bruker, 2000 ▶); data reduction: *SAINT*; program(s) used to solve structure: *SHELXS97* (Sheldrick, 2008 ▶); program(s) used to refine structure: *SHELXL97* (Sheldrick, 2008 ▶); molecular graphics: *ORTEP-3 for Windows* (Farrugia, 2012 ▶) and *PLATON* (Spek, 2009 ▶); software used to prepare material for publication: *SHELXL97*.

## Supplementary Material

Crystal structure: contains datablock(s) I. DOI: 10.1107/S1600536814000774/tk5286sup1.cif

Structure factors: contains datablock(s) I. DOI: 10.1107/S1600536814000774/tk5286Isup2.hkl

CCDC reference:

Additional supporting information:  crystallographic information; 3D view; checkCIF report

## Figures and Tables

**Table:** 

Pd1—P1	2.2676 (16)
Pd1—P2	2.2834 (16)
Pd1—Br1	2.4604 (8)
Pd1—Br2	2.4712 (8)

**Table:** 

P1—Pd1—P2	94.32 (6)
Br1—Pd1—Br2	89.15 (3)

**Table 2:** Hydrogen-bond geometry (Å, °)

*D*—H⋯*A*	*D*—H	H⋯*A*	*D*⋯*A*	*D*—H⋯*A*
C5—H5⋯Br2^i^	0.95	2.91	3.776 (7)	153
C14—H14*A*⋯Br1^ii^	0.99	2.93	3.617 (6)	128

Symmetry codes: (i) 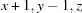; (ii) 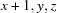.

**Fig. 1.**
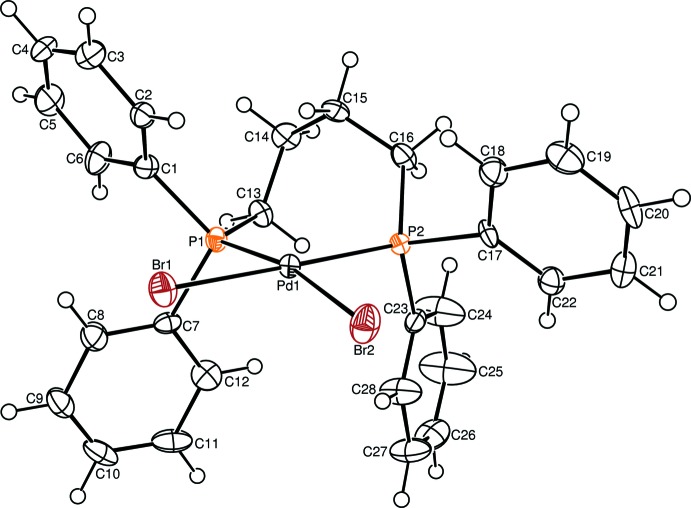
A view of the molecular structure of the title complex, with displacement ellipsoids drawn at the 40% probability level and the atom numbering.

**Fig. 2.**
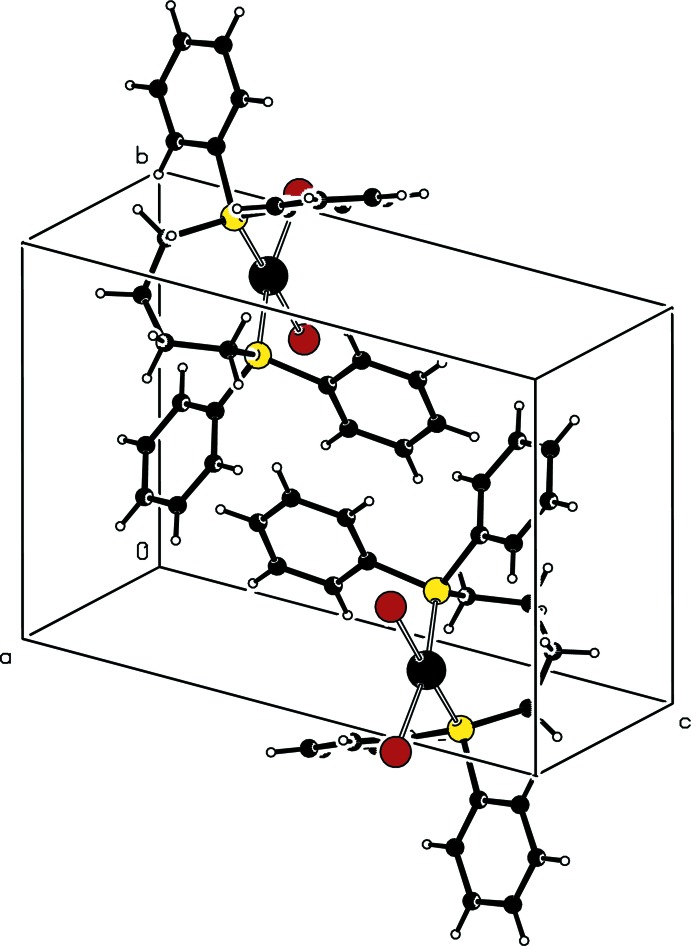
A view of the unit-cell contents of the title complex.

## supplementary crystallographic information

### 2.1. Synthesis and crystallization

To a solution of K_2_PdBr_4_ (0.2526 g, 0.501 mmol) in MeOH (20 ml) was added 1,4-bis­(di­phenyl­phosphanyl)butane (0.2142 g, 0.502 mmol) followed by stirring for 3 h at room temperature. The formed precipitate was separated by filtration, washed with H_2_O and acetone, and dried at 323 K, to give a yellow powder (0.1412 g). Crystals were obtained by slow evaporation from its CH_3_NO_2_ solution held at room temperature.

### 2.2. Refinement

The H atoms were positioned geometrically and allowed to ride on their respective parent atoms: C—H = 0.95 Å (CH) or 0.99 Å (CH_2_) and *U*_iso_(H) = 1.2*U*_eq_(C). The highest peak (1.13 e Å^-3^) and the deepest hole (-0.96 e Å^-3^) in the difference Fourier map are located 1.14 and 0.58 Å, respectively, from the Br1 atom. A large number of reflections were omitted from the final cycles of refinement owing to poor agreement.

### 3. Results and discussion

Crystal structures of the related chlorido Pd^II^ and Pt^II^ complexes, [PdCl_2_(dppb)] (dppb = 1,4-bis­(di­phenyl­phosphanyl)butane, C_28_H_28_P_2_) (Makhaev *et al.*, 1996) and [PtCl_2_(dppb)] (Deacon *et al.*, 2005) have been investigated previously.

The Pd^II^ ion in the title complex, [PdBr_2_(dppb)], has a distorted *cis*-Br_2_P_2_ square-planar coordination geometry defined by two P atoms from the chelating dppb ligand and two Br^-^ anions (Fig. 1). The Pd—P and Pd—Br bond lengths are nearly equivalent, respectively (Table 1). In the crystal, the four phenyl rings are inclined to the least-squares plane of the PdBr_2_P_2_ unit [maximum deviation = 0.1294 (7) Å], making dihedral angles of 66.3 (2) (C1–C6), 87.2 (2) (C7–C12), 68.8 (2) (C17–C22) and 86.8 (2)° (C23–C28). The torsion angle for the four atoms within the butyl­ene chain indicates that the chain is approximately in the *gauche* conformation [C13—C14—C15—C16 = 57.0 (8)°]. The complex molecules are stacked in columns along the *a* axis. In the columns, numerous intra- and inter­molecular π—π inter­actions between the benzene rings are present, the shortest ring centroid-centroid distance being 4.598 (5) Å (Fig. 2). Inter­molecular C—H···Br hydrogen bonds further stabilize the crystal structure (Table 2).

### Crystal data

**Table:**

[PdBr_2_(C_28_H_28_P_2_)]	*Z* = 2
*M**_r_* = 692.66	*F*(000) = 684
Triclinic, *P*1	*D*_x_ = 1.786 Mg m^−^^3^
Hall symbol: -P 1	Mo *K*α radiation, λ = 0.71073 Å
*a* = 8.7481 (5) Å	Cell parameters from 3866 reflections
*b* = 10.7677 (6) Å	θ = 2.4–26.0°
*c* = 14.4789 (8) Å	µ = 3.96 mm^−^^1^
α = 87.203 (1)°	*T* = 200 K
β = 79.389 (1)°	Block, yellow
γ = 73.929 (1)°	0.06 × 0.04 × 0.01 mm
*V* = 1288.15 (13) Å^3^	

### Data collection

**Table:**

Bruker SMART 1000 CCD diffractometer	4961 independent reflections
Radiation source: fine-focus sealed tube	3847 reflections with *I* > 2σ(*I*)
Graphite monochromator	*R*_int_ = 0.024
φ and ω scans	θ_max_ = 26.0°, θ_min_ = 1.4°
Absorption correction: multi-scan (*SADABS*; Bruker, 2000)	*h* = −8→10
*T*_min_ = 0.859, *T*_max_ = 1.000	*k* = −12→13
8009 measured reflections	*l* = −17→17

### Refinement

**Table:**

Refinement on *F*^2^	Primary atom site location: structure-invariant direct methods
Least-squares matrix: full	Secondary atom site location: difference Fourier map
*R*[*F*^2^ > 2σ(*F*^2^)] = 0.045	Hydrogen site location: inferred from neighbouring sites
*wR*(*F*^2^) = 0.120	H-atom parameters constrained
*S* = 1.08	*w* = 1/[σ^2^(*F*_o_^2^) + (0.0398*P*)^2^ + 6.4613*P*] where *P* = (*F*_o_^2^ + 2*F*_c_^2^)/3
4961 reflections	(Δ/σ)_max_ < 0.001
298 parameters	Δρ_max_ = 1.13 e Å^−^^3^
0 restraints	Δρ_min_ = −0.96 e Å^−^^3^

### Special details

**Table:**

Geometry. All e.s.d.'s (except the e.s.d. in the dihedral angle between two l.s. planes) are estimated using the full covariance matrix. The cell e.s.d.'s are taken into account individually in the estimation of e.s.d.'s in distances, angles and torsion angles; correlations between e.s.d.'s in cell parameters are only used when they are defined by crystal symmetry. An approximate (isotropic) treatment of cell e.s.d.'s is used for estimating e.s.d.'s involving l.s. planes.
Refinement. Refinement of *F*^2^ against ALL reflections. The weighted *R*-factor *wR* and goodness of fit *S* are based on *F*^2^, conventional *R*-factors *R* are based on *F*, with *F* set to zero for negative *F*^2^. The threshold expression of *F*^2^ > σ(*F*^2^) is used only for calculating *R*-factors(gt) *etc*. and is not relevant to the choice of reflections for refinement. *R*-factors based on *F*^2^ are statistically about twice as large as those based on *F*, and *R*- factors based on ALL data will be even larger.

### Fractional atomic coordinates and isotropic or equivalent isotropic displacement parameters (Å^2^)

**Table:**

	*x*	*y*	*z*	*U*_iso_*/*U*_eq_	
Pd1	0.11465 (5)	0.83898 (4)	0.24341 (3)	0.01959 (13)	
Br1	−0.02105 (8)	0.66561 (7)	0.27646 (5)	0.03468 (19)	
Br2	−0.14937 (9)	0.99359 (7)	0.23280 (6)	0.0411 (2)	
P1	0.33484 (19)	0.69181 (14)	0.28150 (11)	0.0216 (3)	
P2	0.24181 (19)	0.99889 (14)	0.21058 (11)	0.0204 (3)	
C1	0.4032 (7)	0.5406 (6)	0.2161 (4)	0.0241 (13)	
C2	0.3474 (8)	0.5242 (6)	0.1356 (4)	0.0272 (14)	
H2	0.2631	0.5902	0.1155	0.033*	
C3	0.4149 (9)	0.4111 (7)	0.0844 (5)	0.0374 (17)	
H3	0.3777	0.3997	0.0286	0.045*	
C4	0.5372 (10)	0.3136 (7)	0.1143 (5)	0.0409 (18)	
H4	0.5824	0.2358	0.0791	0.049*	
C5	0.5920 (9)	0.3292 (7)	0.1931 (6)	0.0411 (18)	
H5	0.6771	0.2631	0.2124	0.049*	
C6	0.5244 (9)	0.4414 (6)	0.2458 (5)	0.0371 (17)	
H6	0.5607	0.4508	0.3023	0.044*	
C7	0.2871 (7)	0.6503 (6)	0.4044 (4)	0.0238 (13)	
C8	0.2367 (9)	0.5413 (7)	0.4322 (5)	0.0345 (16)	
H8	0.2323	0.4829	0.3862	0.041*	
C9	0.1921 (9)	0.5164 (7)	0.5277 (5)	0.0372 (17)	
H9	0.1606	0.4401	0.5467	0.045*	
C10	0.1941 (9)	0.6014 (8)	0.5925 (5)	0.0406 (18)	
H10	0.1619	0.5844	0.6571	0.049*	
C11	0.2413 (9)	0.7116 (8)	0.5679 (5)	0.044 (2)	
H11	0.2400	0.7705	0.6150	0.053*	
C12	0.2918 (9)	0.7370 (7)	0.4721 (5)	0.0378 (17)	
H12	0.3282	0.8114	0.4540	0.045*	
C13	0.5229 (7)	0.7372 (6)	0.2743 (4)	0.0260 (14)	
H13A	0.4977	0.8243	0.3022	0.031*	
H13B	0.5929	0.6759	0.3124	0.031*	
C14	0.6160 (7)	0.7384 (7)	0.1750 (5)	0.0299 (15)	
H14A	0.7091	0.7731	0.1770	0.036*	
H14B	0.6599	0.6482	0.1521	0.036*	
C15	0.5194 (8)	0.8167 (6)	0.1050 (4)	0.0276 (14)	
H15A	0.5917	0.8128	0.0433	0.033*	
H15B	0.4337	0.7762	0.0974	0.033*	
C16	0.4422 (8)	0.9557 (6)	0.1317 (5)	0.0299 (14)	
H16A	0.4311	1.0058	0.0729	0.036*	
H16B	0.5183	0.9856	0.1620	0.036*	
C17	0.1346 (8)	1.1362 (6)	0.1468 (4)	0.0252 (14)	
C18	0.1042 (9)	1.1129 (7)	0.0585 (5)	0.0328 (15)	
H18	0.1355	1.0271	0.0348	0.039*	
C19	0.0289 (9)	1.2140 (8)	0.0056 (5)	0.0402 (18)	
H19	0.0089	1.1982	−0.0545	0.048*	
C20	−0.0162 (9)	1.3367 (7)	0.0406 (5)	0.0385 (18)	
H20	−0.0684	1.4060	0.0043	0.046*	
C21	0.0120 (9)	1.3630 (7)	0.1278 (5)	0.0381 (17)	
H21	−0.0202	1.4491	0.1510	0.046*	
C22	0.0887 (8)	1.2603 (6)	0.1812 (5)	0.0303 (15)	
H22	0.1090	1.2765	0.2411	0.036*	
C23	0.2624 (8)	1.0629 (5)	0.3199 (4)	0.0237 (13)	
C24	0.3971 (11)	1.0970 (11)	0.3321 (6)	0.066 (3)	
H24	0.4852	1.0889	0.2813	0.079*	
C25	0.4052 (12)	1.1432 (12)	0.4179 (7)	0.082 (4)	
H25	0.4983	1.1682	0.4252	0.099*	
C26	0.2834 (10)	1.1533 (8)	0.4913 (6)	0.047 (2)	
H26	0.2932	1.1811	0.5507	0.057*	
C27	0.1464 (10)	1.1238 (8)	0.4807 (5)	0.050 (2)	
H27	0.0574	1.1353	0.5312	0.060*	
C28	0.1389 (9)	1.0763 (8)	0.3940 (5)	0.046 (2)	
H28	0.0449	1.0527	0.3868	0.055*	

### Atomic displacement parameters (Å^2^)

**Table:**

	*U*^11^	*U*^22^	*U*^33^	*U*^12^	*U*^13^	*U*^23^
Pd1	0.0200 (2)	0.0182 (2)	0.0209 (2)	−0.00528 (18)	−0.00436 (18)	0.00072 (17)
Br1	0.0327 (4)	0.0324 (4)	0.0422 (4)	−0.0135 (3)	−0.0096 (3)	0.0062 (3)
Br2	0.0281 (4)	0.0293 (4)	0.0630 (5)	−0.0029 (3)	−0.0091 (3)	0.0027 (3)
P1	0.0231 (8)	0.0191 (7)	0.0231 (8)	−0.0048 (6)	−0.0079 (6)	0.0026 (6)
P2	0.0231 (8)	0.0182 (7)	0.0192 (8)	−0.0064 (6)	−0.0013 (6)	0.0006 (6)
C1	0.022 (3)	0.024 (3)	0.024 (3)	−0.001 (3)	−0.005 (3)	0.001 (2)
C2	0.027 (3)	0.023 (3)	0.028 (3)	−0.003 (3)	−0.001 (3)	0.002 (3)
C3	0.044 (4)	0.033 (4)	0.034 (4)	−0.009 (3)	−0.006 (3)	−0.005 (3)
C4	0.056 (5)	0.025 (3)	0.037 (4)	−0.014 (3)	0.010 (4)	−0.013 (3)
C5	0.030 (4)	0.031 (4)	0.051 (5)	0.007 (3)	−0.002 (3)	0.005 (3)
C6	0.039 (4)	0.024 (3)	0.047 (4)	−0.001 (3)	−0.018 (3)	0.000 (3)
C7	0.021 (3)	0.028 (3)	0.019 (3)	−0.001 (3)	−0.006 (2)	−0.004 (2)
C8	0.043 (4)	0.032 (4)	0.029 (4)	−0.010 (3)	−0.009 (3)	0.005 (3)
C9	0.039 (4)	0.038 (4)	0.034 (4)	−0.011 (3)	−0.005 (3)	0.013 (3)
C10	0.033 (4)	0.059 (5)	0.024 (4)	−0.009 (4)	−0.001 (3)	0.016 (3)
C11	0.043 (4)	0.069 (5)	0.018 (3)	−0.008 (4)	−0.007 (3)	−0.011 (3)
C12	0.037 (4)	0.043 (4)	0.036 (4)	−0.013 (3)	−0.012 (3)	0.002 (3)
C13	0.026 (3)	0.030 (3)	0.026 (3)	−0.010 (3)	−0.012 (3)	0.008 (3)
C14	0.012 (3)	0.037 (4)	0.037 (4)	−0.001 (3)	−0.004 (3)	0.001 (3)
C15	0.022 (3)	0.036 (4)	0.023 (3)	−0.009 (3)	0.004 (3)	0.001 (3)
C16	0.030 (4)	0.032 (3)	0.028 (3)	−0.012 (3)	−0.001 (3)	0.007 (3)
C17	0.033 (4)	0.021 (3)	0.022 (3)	−0.010 (3)	−0.007 (3)	0.012 (2)
C18	0.042 (4)	0.031 (4)	0.028 (4)	−0.012 (3)	−0.010 (3)	0.002 (3)
C19	0.043 (4)	0.056 (5)	0.029 (4)	−0.020 (4)	−0.013 (3)	0.003 (3)
C20	0.042 (4)	0.035 (4)	0.047 (4)	−0.020 (3)	−0.019 (4)	0.025 (3)
C21	0.041 (4)	0.023 (3)	0.051 (5)	−0.007 (3)	−0.012 (4)	0.004 (3)
C22	0.036 (4)	0.028 (3)	0.029 (4)	−0.013 (3)	−0.005 (3)	0.000 (3)
C23	0.030 (3)	0.017 (3)	0.024 (3)	−0.002 (3)	−0.007 (3)	−0.002 (2)
C24	0.048 (5)	0.123 (9)	0.043 (5)	−0.054 (6)	0.001 (4)	−0.022 (5)
C25	0.062 (6)	0.152 (11)	0.058 (6)	−0.071 (7)	0.001 (5)	−0.037 (7)
C26	0.059 (5)	0.049 (5)	0.036 (4)	−0.007 (4)	−0.021 (4)	−0.014 (4)
C27	0.048 (5)	0.068 (6)	0.033 (4)	−0.019 (4)	0.004 (4)	−0.023 (4)
C28	0.036 (4)	0.073 (6)	0.036 (4)	−0.029 (4)	0.001 (3)	−0.018 (4)

### Geometric parameters (Å, º)

**Table:**

Pd1—P1	2.2676 (16)	C13—H13A	0.9900
Pd1—P2	2.2834 (16)	C13—H13B	0.9900
Pd1—Br1	2.4604 (8)	C14—C15	1.514 (9)
Pd1—Br2	2.4712 (8)	C14—H14A	0.9900
P1—C7	1.814 (6)	C14—H14B	0.9900
P1—C1	1.819 (6)	C15—C16	1.499 (9)
P1—C13	1.826 (6)	C15—H15A	0.9900
P2—C23	1.816 (6)	C15—H15B	0.9900
P2—C17	1.827 (6)	C16—H16A	0.9900
P2—C16	1.860 (7)	C16—H16B	0.9900
C1—C2	1.381 (9)	C17—C22	1.374 (9)
C1—C6	1.395 (9)	C17—C18	1.401 (9)
C2—C3	1.383 (9)	C18—C19	1.383 (10)
C2—H2	0.9500	C18—H18	0.9500
C3—C4	1.392 (10)	C19—C20	1.364 (10)
C3—H3	0.9500	C19—H19	0.9500
C4—C5	1.350 (11)	C20—C21	1.387 (10)
C4—H4	0.9500	C20—H20	0.9500
C5—C6	1.386 (10)	C21—C22	1.403 (9)
C5—H5	0.9500	C21—H21	0.9500
C6—H6	0.9500	C22—H22	0.9500
C7—C8	1.382 (9)	C23—C28	1.357 (9)
C7—C12	1.400 (9)	C23—C24	1.369 (10)
C8—C9	1.399 (9)	C24—C25	1.382 (12)
C8—H8	0.9500	C24—H24	0.9500
C9—C10	1.349 (11)	C25—C26	1.344 (12)
C9—H9	0.9500	C25—H25	0.9500
C10—C11	1.371 (11)	C26—C27	1.358 (11)
C10—H10	0.9500	C26—H26	0.9500
C11—C12	1.414 (10)	C27—C28	1.397 (10)
C11—H11	0.9500	C27—H27	0.9500
C12—H12	0.9500	C28—H28	0.9500
C13—C14	1.518 (9)		
			
P1—Pd1—P2	94.32 (6)	C14—C13—H13B	108.8
P1—Pd1—Br1	85.65 (4)	P1—C13—H13B	108.8
P2—Pd1—Br1	179.19 (5)	H13A—C13—H13B	107.7
P1—Pd1—Br2	169.15 (5)	C15—C14—C13	115.2 (5)
P2—Pd1—Br2	91.00 (4)	C15—C14—H14A	108.5
Br1—Pd1—Br2	89.15 (3)	C13—C14—H14A	108.5
C7—P1—C1	106.6 (3)	C15—C14—H14B	108.5
C7—P1—C13	103.2 (3)	C13—C14—H14B	108.5
C1—P1—C13	101.9 (3)	H14A—C14—H14B	107.5
C7—P1—Pd1	108.0 (2)	C16—C15—C14	114.5 (6)
C1—P1—Pd1	116.4 (2)	C16—C15—H15A	108.6
C13—P1—Pd1	119.3 (2)	C14—C15—H15A	108.6
C23—P2—C17	106.4 (3)	C16—C15—H15B	108.6
C23—P2—C16	109.1 (3)	C14—C15—H15B	108.6
C17—P2—C16	100.2 (3)	H15A—C15—H15B	107.6
C23—P2—Pd1	109.2 (2)	C15—C16—P2	118.6 (4)
C17—P2—Pd1	114.5 (2)	C15—C16—H16A	107.7
C16—P2—Pd1	116.8 (2)	P2—C16—H16A	107.7
C2—C1—C6	119.5 (6)	C15—C16—H16B	107.7
C2—C1—P1	122.4 (5)	P2—C16—H16B	107.7
C6—C1—P1	118.0 (5)	H16A—C16—H16B	107.1
C1—C2—C3	119.6 (6)	C22—C17—C18	119.8 (6)
C1—C2—H2	120.2	C22—C17—P2	122.2 (5)
C3—C2—H2	120.2	C18—C17—P2	118.0 (5)
C2—C3—C4	120.2 (7)	C19—C18—C17	120.4 (6)
C2—C3—H3	119.9	C19—C18—H18	119.8
C4—C3—H3	119.9	C17—C18—H18	119.8
C5—C4—C3	120.4 (6)	C20—C19—C18	119.2 (7)
C5—C4—H4	119.8	C20—C19—H19	120.4
C3—C4—H4	119.8	C18—C19—H19	120.4
C4—C5—C6	120.1 (7)	C19—C20—C21	121.8 (6)
C4—C5—H5	119.9	C19—C20—H20	119.1
C6—C5—H5	119.9	C21—C20—H20	119.1
C5—C6—C1	120.2 (7)	C20—C21—C22	118.9 (6)
C5—C6—H6	119.9	C20—C21—H21	120.6
C1—C6—H6	119.9	C22—C21—H21	120.6
C8—C7—C12	119.8 (6)	C17—C22—C21	119.9 (6)
C8—C7—P1	122.0 (5)	C17—C22—H22	120.0
C12—C7—P1	118.1 (5)	C21—C22—H22	120.0
C7—C8—C9	120.3 (6)	C28—C23—C24	118.1 (6)
C7—C8—H8	119.8	C28—C23—P2	118.4 (5)
C9—C8—H8	119.8	C24—C23—P2	123.5 (6)
C10—C9—C8	119.6 (7)	C23—C24—C25	120.2 (8)
C10—C9—H9	120.2	C23—C24—H24	119.9
C8—C9—H9	120.2	C25—C24—H24	119.9
C9—C10—C11	121.9 (6)	C26—C25—C24	121.0 (8)
C9—C10—H10	119.1	C26—C25—H25	119.5
C11—C10—H10	119.1	C24—C25—H25	119.5
C10—C11—C12	119.6 (7)	C25—C26—C27	120.1 (7)
C10—C11—H11	120.2	C25—C26—H26	119.9
C12—C11—H11	120.2	C27—C26—H26	119.9
C7—C12—C11	118.7 (7)	C26—C27—C28	118.7 (7)
C7—C12—H12	120.6	C26—C27—H27	120.7
C11—C12—H12	120.6	C28—C27—H27	120.7
C14—C13—P1	114.0 (4)	C23—C28—C27	121.7 (7)
C14—C13—H13A	108.8	C23—C28—H28	119.1
P1—C13—H13A	108.8	C27—C28—H28	119.1
			
P2—Pd1—P1—C7	116.6 (2)	C9—C10—C11—C12	−1.0 (11)
Br1—Pd1—P1—C7	−64.2 (2)	C8—C7—C12—C11	−1.2 (10)
Br2—Pd1—P1—C7	−2.6 (4)	P1—C7—C12—C11	174.5 (5)
P2—Pd1—P1—C1	−123.6 (2)	C10—C11—C12—C7	2.1 (11)
Br1—Pd1—P1—C1	55.6 (2)	C7—P1—C13—C14	162.9 (5)
Br2—Pd1—P1—C1	117.2 (3)	C1—P1—C13—C14	52.4 (5)
P2—Pd1—P1—C13	−0.7 (2)	Pd1—P1—C13—C14	−77.4 (5)
Br1—Pd1—P1—C13	178.5 (2)	P1—C13—C14—C15	52.1 (7)
Br2—Pd1—P1—C13	−119.9 (3)	C13—C14—C15—C16	57.0 (8)
P1—Pd1—P2—C23	−75.1 (2)	C14—C15—C16—P2	−85.3 (6)
Br1—Pd1—P2—C23	−163 (3)	C23—P2—C16—C15	115.9 (5)
Br2—Pd1—P2—C23	95.4 (2)	C17—P2—C16—C15	−132.6 (5)
P1—Pd1—P2—C17	165.7 (2)	Pd1—P2—C16—C15	−8.3 (6)
Br1—Pd1—P2—C17	77 (3)	C23—P2—C17—C22	1.6 (6)
Br2—Pd1—P2—C17	−23.8 (2)	C16—P2—C17—C22	−111.9 (6)
P1—Pd1—P2—C16	49.1 (2)	Pd1—P2—C17—C22	122.3 (5)
Br1—Pd1—P2—C16	−39 (3)	C23—P2—C17—C18	179.4 (5)
Br2—Pd1—P2—C16	−140.4 (2)	C16—P2—C17—C18	65.9 (6)
C7—P1—C1—C2	133.2 (5)	Pd1—P2—C17—C18	−59.9 (6)
C13—P1—C1—C2	−118.9 (6)	C22—C17—C18—C19	0.3 (10)
Pd1—P1—C1—C2	12.7 (6)	P2—C17—C18—C19	−177.6 (6)
C7—P1—C1—C6	−51.2 (6)	C17—C18—C19—C20	−0.4 (11)
C13—P1—C1—C6	56.7 (6)	C18—C19—C20—C21	0.3 (12)
Pd1—P1—C1—C6	−171.7 (5)	C19—C20—C21—C22	−0.1 (11)
C6—C1—C2—C3	−1.5 (10)	C18—C17—C22—C21	−0.1 (10)
P1—C1—C2—C3	174.1 (5)	P2—C17—C22—C21	177.6 (5)
C1—C2—C3—C4	0.7 (10)	C20—C21—C22—C17	0.1 (11)
C2—C3—C4—C5	−0.6 (11)	C17—P2—C23—C28	85.7 (6)
C3—C4—C5—C6	1.3 (12)	C16—P2—C23—C28	−167.1 (6)
C4—C5—C6—C1	−2.2 (12)	Pd1—P2—C23—C28	−38.4 (6)
C2—C1—C6—C5	2.2 (11)	C17—P2—C23—C24	−95.2 (7)
P1—C1—C6—C5	−173.5 (6)	C16—P2—C23—C24	12.1 (8)
C1—P1—C7—C8	−28.5 (6)	Pd1—P2—C23—C24	140.7 (7)
C13—P1—C7—C8	−135.4 (6)	C28—C23—C24—C25	0.2 (15)
Pd1—P1—C7—C8	97.3 (5)	P2—C23—C24—C25	−178.9 (8)
C1—P1—C7—C12	155.9 (5)	C23—C24—C25—C26	1.2 (18)
C13—P1—C7—C12	48.9 (6)	C24—C25—C26—C27	−3.2 (17)
Pd1—P1—C7—C12	−78.3 (5)	C25—C26—C27—C28	3.5 (14)
C12—C7—C8—C9	−0.7 (10)	C24—C23—C28—C27	0.3 (13)
P1—C7—C8—C9	−176.3 (5)	P2—C23—C28—C27	179.4 (7)
C7—C8—C9—C10	1.9 (11)	C26—C27—C28—C23	−2.1 (13)
C8—C9—C10—C11	−1.0 (11)		

### Hydrogen-bond geometry (Å, º)

**Table:**

*D*—H···*A*	*D*—H	H···*A*	*D*···*A*	*D*—H···*A*
C5—H5···Br2^i^	0.95	2.91	3.776 (7)	153
C14—H14*A*···Br1^ii^	0.99	2.93	3.617 (6)	128

Symmetry codes: (i) *x*+1, *y*−1, *z*; (ii) *x*+1, *y*, *z*.
